# Comparison of Glutathione Nanoparticles, CoEnzyme Q10, and Fish Oil for Prevention of Oxygen-Induced Retinopathy in Neonatal Rats

**DOI:** 10.3390/ph17030381

**Published:** 2024-03-17

**Authors:** Sidra Bashir, Charles L. Cai, Matthew Marcelino, Jacob V. Aranda, Kay D. Beharry

**Affiliations:** 1 Department of Pediatrics, Division of Neonatal-Perinatal Medicine, State University of New York Downstate Health Sciences University, Brooklyn, NY 11203, USA; sidra.bashir@downstate.edu (S.B.); charles.cai@downstate.edu (C.L.C.); jacob.aranda@downstate.edu (J.V.A.); 2 Medical School, State University of New York Downstate Health Sciences University, Brooklyn, NY 11203, USA; matthew.marcelino@downstate.edu; 3 Department of Ophthalmology, State University of New York Downstate Health Sciences University, Brooklyn, NY 11203, USA; 4 SUNY Eye Institute, Brooklyn, NY 11203, USA

**Keywords:** coenzyme Q10, fish oil, glutathione nanoparticles, neonatal intermittent hypoxia, notch signaling, oxygen-induced retinopathy

## Abstract

Notch ligands and receptors are important for cell specification and angiogenesis, but their role in oxygen-induced retinopathy (OIR) is not well studied. Delta-like ligand (DLL)-4/Notch inhibits angiogenesis, while Jagged-1/Notch promotes angiogenesis. We tested the hypothesis that early supplementation with antioxidants and/or fish oil curtails severe OIR by inducing DLL-4/Notch and reducing Jagged-1/Notch. Newborn rats were exposed to brief intermittent hypoxia (IH) during hyperoxia, during which they received daily oral supplements of (1) fish oil, (2) coenzyme Q10 (CoQ10) in olive oil (OO), (3) glutathione nanoparticles (nGSH), (4) fish oil + CoQ10, or (5) OO (controls) from birth (P0) to P14. At P14, the pups were placed in room air (RA) until P21, with no further treatment. Oxidative stress, apoptosis, ocular histopathology, and Notch signaling were assessed. Neonatal IH resulted in severe retinal damage consistent with retinopathy of prematurity (ROP). Retinal damage was associated with induced oxidative stress and Jagged-1/Notch signaling, as well as reduced DLL-4/Notch signaling. All treatments reversed these outcomes, but nGSH produced the most beneficial outcomes. Severe OIR promoted the induction of Jagged-1/Notch and curtailed DLL-4/Notch, which was an effect that could be reversed with nGSH supplementation. These findings may indicate a potential alternate pathway for ROP treatment and/or prevention.

## 1. Introduction

Extremely low gestational age neonates (ELGANs) who experience frequent desaturations and/or apneas are at high risk for oxygen radical diseases of newborn, including retinopathy of prematurity (ROP). ROP remains the leading cause of childhood blindness worldwide [1,2], accounting for 40% of cases [3] and causing significant health burdens in the form of childhood ophthalmic complications, such as glaucoma, strabismus, amblyopia, refractive errors, cataracts, nystagmus, and retinal detachment [4,5,6], as well as the huge financial and emotional stress to the families [7,8,9]. ROP is a neovascular disease that occurs as a result of the disruption of retinal vascular development due to exposure to oxygen therapy. Hyperoxia suppresses growth factors, such as vascular endothelial growth factor (VEGF), which is a potent endothelial cell (EC) mitogen, with resulting vaso-obliteration and reduced oxygen in the affected areas. The resulting hypoxia then induces VEGF via the activation of hypoxia-inducible factor (HIF)_1α_, causing vascular overgrowth or neovascularization, which can induce fibrotic scarring in the retina, vitreous, and lens [10]. While early birth, low birth weight, and postnatal hyperoxia exposure are the universally accepted disease predictors [3,11,12,13], neonatal intermittent hypoxia (IH) followed by reoxygenation in normoxia or hyperoxia with supplemental oxygen has emerged as one of the major factors associated with severe ROP in ELGANs requiring oxygen therapy [14,15,16], and in animal models of oxygen-induced retinopathy (OIR) [17,18].

Retinal angiogenesis is regulated by complex interactions between local and systemically produced growth factors, particularly VEGF [19,20]. VEGF expression is under the strong transcriptional control of environmental influences, including hypoxia. The differential response to VEGF is the migration of tip cells to direct the vascular sprout and proliferation of stalk cells [20,21], with the simultaneous induction of Notch signaling [22]. The Notch signaling pathway appears to be similarly important by playing a fine-tuning function in physiological and pathological angiogenesis, which subsequently promotes the differentiation of the tip versus stalk cells and inhibits VEGF-induced EC proliferation [22,23,24,25,26]. Recent studies showed that the delta-like ligand (DLL)-4 expressed in the tip cells activates Notch-1 in adjacent stalk cells, leading to the upregulation of Jagged-1 [26]. DLL-4/Notch-associated transduction reduces the expression of VEGF receptor (VEGFR)-2, lowering the ECs sensitivity to VEGF, with the restriction of tip cells and excessive sprouting, whereas Jagged-1 antagonizes DLL-4’s ability to activate Notch-1 in the tip cells, creating a negative feedback loop, thus acting as a proangiogenic factor [27].

Due to significant limitations, side-effects, and systemic concerns regarding current treatment modalities [28,29,30,31,32,33], several preventative therapies targeting mechanisms within the first phase (vaso-obliteration) of ROP have been explored. One of the most promising and least harmful of which is fish oil [34,35,36,37,38,39] and combination fish oil with antioxidants [40,41,42], which were shown to decrease neovascularization in humans and animal models. However, the effects of fish oil and/or antioxidants on Notch ligands and receptors were not previously studied. Extensive research regarding the role of Notch signaling pathways in ocular angiogenesis [23,43,44] has highlighted the importance of Notch ligands as potential therapeutic targets. However, studies involving the role of Notch signaling in the development of ROP are minimal. To this end, we conducted a series of experiments to test the hypothesis that IH-induced retinopathy is associated with a reduction in DLL-4/Notch and an induction of Jagged-1/Notch, and that supplementation with antioxidants and/or fish oil reverses these effects and reduces the severity of OIR. Our primary outcome was retinal vascular and astrocyte integrity, and our secondary outcomes were retinal histopathology and the levels of angiogenesis biomarkers.

## 2. Results

Animals were exposed to neonatal IH from postnatal day 0 (P0) to P14, during which they received daily oral olive oil (OO), coenzyme Q10 (CoQ10), fish oil (FO), glutathione nanoparticles (nGSH), or the CoQ10 + FO combination. At P14, the pups were placed in RA for recovery/reoxygenation until P21. Room air (RA) littermates were raised in 21% O_2_ until P21 with similar treatment. A representation of the experimental design is presented in the supplementary figures (Figure S1). All samples were collected at P21. Adenosine diphosphatase (ADPase) and glial fibrillary acidic protein (GFAP)/isolectin B4 staining of the retinas were conducted on the same day of sample collection at P21. Histopathological assessments were conducted 7 days after the sample collection. For all other assays, samples were snap frozen in liquid nitrogen on the day of harvest and stored at −80 °C until analysis.

### 2.1. Retinal Vasculature

**Figure 1:**
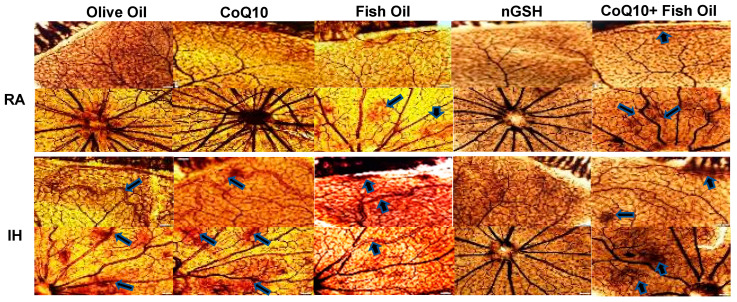
Representative ADPase-stained retinal flatmounts showing retinal vascular architecture at P21. Composite panels are retinal periphery (**upper**) and optic disk (**lower**). The upper composite panels are the room air (RA)-exposed animals, and the lower composite panels are the intermittent hypoxia (IH)-exposed animals. Images show 10× magnification; the scale bar is 100 µM. Animals exposed to neonatal IH showed vascular abundance at the periphery, vascular tortuosity, hemorrhage, enlarged vessels, and vascular tufts (arrows).

Representative ADPase-stained (brown) retinas from the RA controls and IH groups are presented in Figure 1. Images show 10× magnification (scale bar is 100 µm). Composite images for each group in RA or IH include retinal vasculature at the periphery (upper panel) and retinal vasculature at the optic disk (lower panel). In RA, supplementation with fish oil resulted in punctate hemorrhage at the optic disk, while supplementation with the CoQ10 + FO combination resulted in vascular abundance at the periphery, as well as tortuous vessels with some hemorrhage at the optic disk (arrows). In contrast, exposure to neonatal IH showed significant vascular abnormalities in all groups, except for the glutathione nanoparticles (nGSH). These abnormalities included enlarged tortuous vessels, vascular tufts, hemorrhage, and abundant disorganized vasculature (arrows). A quantitative analysis showed significant reductions in arterial diameter with nGSH and CoQ10 + FO supplementation in RA and neonatal IH. In neonatal IH, all treatments reduced the vascular tortuosity compared with OO (Table 1).

### 2.2. Retinal Astrocytes

**Figure 2:**
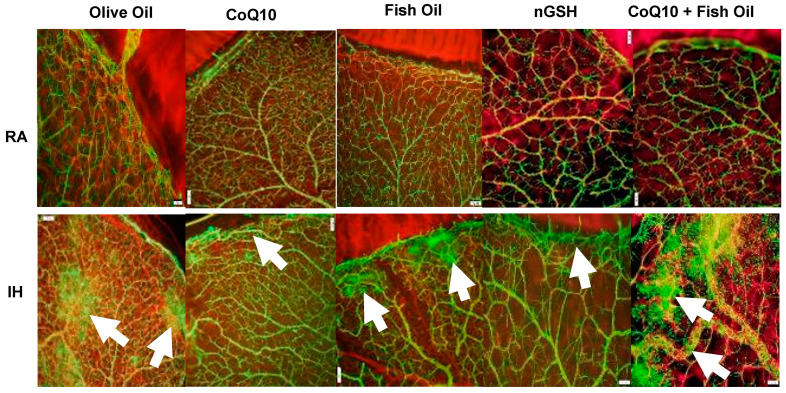
Representative GFAP and GS–isolectin double-stained retinal flatmounts showing astrocytic template (green) and retinal vasculature (red) at the periphery. Images show 10× magnification; scale bar is 100 µM. Images show abnormalities in the astrocytic template with olive oil, fish oil, and CoQ10 + fish oil in neonatal IH (arrows).

During retinal development, retinal astrocytes, which are predominantly located in the nuclear fiber layer (NFL)/ganglion cell layer (GCL) layer, are important for normal vascularization. They emerge from the optic disc and lay the foundation for the vascular template. The effect of neonatal IH with supplementation on the retinal astrocyte template is presented in Figure 2. GFAP expression is a marker for the activation of astrocytes. GFAP is represented by the green stain, and isolectin B4, which was the marker for blood vessels, is represented by the red stain. Images show 20× magnification (scale bar is 50 µm). The upper panels represent the RA groups and the lower panels represent the IH groups. There was no disturbance in the astrocytic template with any treatment in RA. In contrast, retinas exposed to IH showed major disturbances in the astrocytic template. In particular, the groups treated with OO, FO, and CoQ10 + FO showed significant astrocyte disruptions and possible Müller end foot reactivity (arrows), which may indicate reactive gliosis. The expression of GFAP in the retinal layers (brown stain) is presented in the Supplementary Materials (Figure S2). In RA, a higher expression in GFAP was seen with CoQ10 and reduction with CoQ10 + FO compared with OO. In IH, GFAP was elevated in all groups, but the lowest was seen with CoQ10 and nGSH. The corresponding quantitative analysis results are presented in Table 2.

### 2.3. Retinal Histopathology

**Figure 3:**
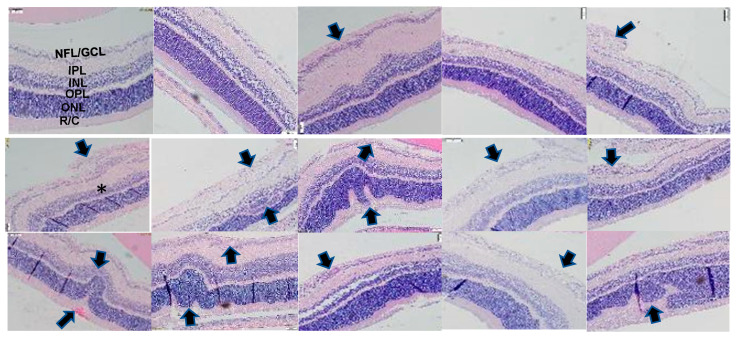
Representative H&E-stained retinas from neonatal rats at P21. The retinal layers were identified in the olive oil group exposed to room air (RA). RA-exposed retinas are represented in the upper panels and IH-exposed retinas are represented in the lower composite panels. Exposure to IH caused retinal endothelial cells to penetrate the inner limiting membrane and migrate into the vitreous fluid (arrows), vitreous condensation and traction (∗), choroidal hemorrhage, retinal folds pulling the retina away from the sclera, and outer nuclear layer damage (arrows). Images show 20× magnification (scale bar is 50 µM).

Representative hematoxylin and eosin (H&E)-stained retinal layers are presented in Figure 3. Images show 20× magnification (scale bar is 50 µm). The layers are labeled in the RA OO group. Composite images are presented for each treatment in IH due to the number of abnormalities. In RA, supplementation with fish oil and CoQ10 + FO resulted in increased numbers of cells and a widening of the NFL/GCL layers compared with the other groups (arrows). In neonatal IH, the group treated with olive oil showed significantly large vessels migrating into the vitreous fluid (arrow), as well as choroidal hemorrhage and invaginations of the ONL and INL, indicating separation from the sclera and retinal detachment (arrow). Treatment with CoQ10, fish oil, and CoQ10 + fish oil also showed large vessels in the NFL/GCL layer and ONL abnormalities (arrows), while nGSH resulted in widening of the NFL/GCL layer (arrows), but the number of ECs were reduced compared with the other groups. Retinal thickness is an important predictor of “plus” disease or stage 3 ROP in preterm infants [45]. Quantitative analysis of the retinal layers is presented in the Supplementary Materials (Table S1). Data showed that the total retinal thickness was significantly elevated with fish oil and to a lesser degree with CoQ10 + fish oil in RA. This was coincident with elevations in the NFL/GCL, IPL, OPL, and R/C layers. In neonatal IH, all treatments resulted in higher numbers of ECs in the NFL/GCL layer, but the levels were the most elevated with OO, FO, and CoQ10 + FO. Overall, the exposure to neonatal IH resulted in increased thicknesses in all layers, but all supplements reduced the retinal layer thickness compared with olive oil.

### 2.4. Notch-1

**Figure 4:**
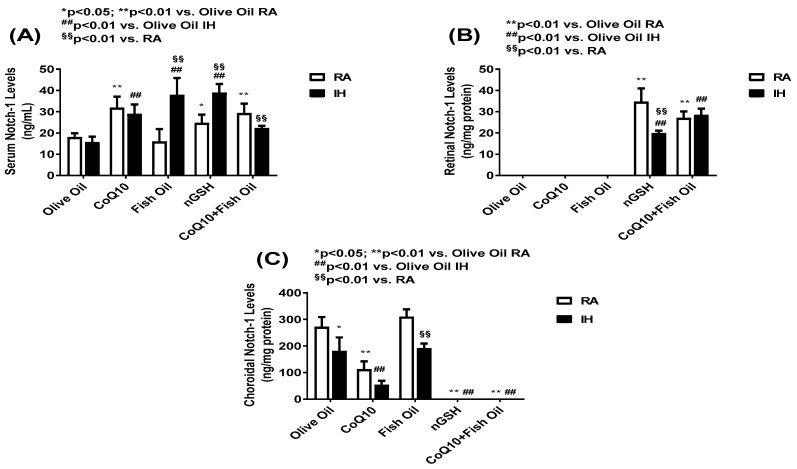
Levels of Notch-1 in the serum (**A**), retina (**B**), and choroid (**C**) in response to neonatal IH with olive oil, CoQ10, fish oil, nGSH, or CoQ10 + fish oil supplementation. Levels in the retinal and choroidal homogenates were standardized using total cellular protein levels. The open bars represent the room air (RA) groups and the solid bars represent the IH groups. Data are expressed as mean ± SD (*n* = 6 samples/group).

Signaling through Notch receptors is important for regulating angiogenesis and vascular differentiation during development and in diverse pathological conditions. Figure 4 shows Notch-1 levels in the serum (panel A), retinal homogenates (panel B), and choroidal homogenates (panel C). In the serum, Notch-1 was increased with CoQ10, nGSH, and the CoQ10 + FO co-treatment in RA, while all treatments increased Notch-1 serum levels in IH (panel A). In contrast, significant elevations were seen in the retina only with nGSH and the CoQ10 + FO co-treatment in RA and IH, while OO, CoQ10, and FO eliminated Notch-1 in the retina (panel B). In the choroid, an opposite response was noted. OO, CoQ10, and FO induced Notch-1, while nGSH and CoQ10 + FO abolished it. In RA, only CoQ10 decreased the choroidal Notch-1 in RA and IH, although lower levels were seen with OO and FO treatments in IH (panel C).

**Figure 5:**
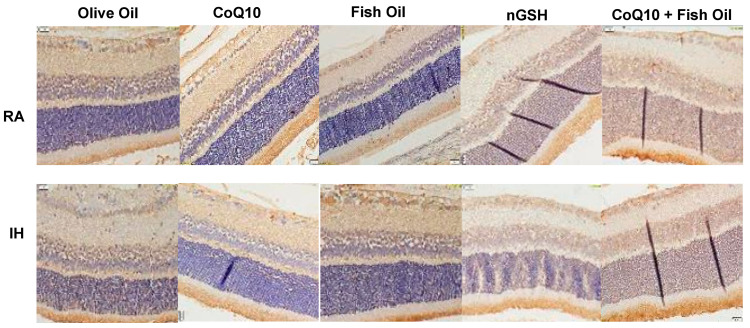
Representative immunoreactivity of Notch-1 (brown) in the retinal layers at P21. Images were counterstained with hematoxylin (blue). RA-exposed retinas are represented in the upper panels and IH-exposed retinas are represented in the lower panels. Images show 40× magnification (scale bar is 20 µM).

Representative Notch-1 expression in the retinal layers (brown stain) is presented in Figure 5). Overall, the expression of Notch-1 was the highest in the NFL/GCL and rod and cone (R/C) layers. Quantitative analysis of the expression intensity, presented in Table 2, shows elevations in RA with all treatments compared with olive oil, and elevations with nGSH and CoQ10 + fish oil in IH.

### 2.5. Notch-4

**Figure 6:**
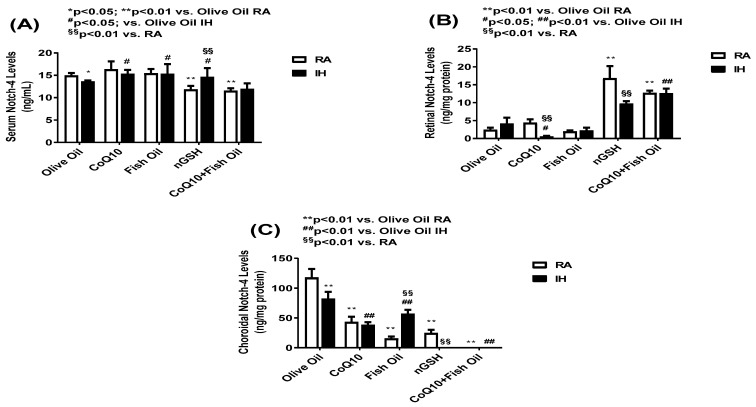
Levels of Notch-4 in the serum (**A**), retina (**B**), and choroid (**C**) in response to neonatal IH with olive oil, CoQ10, fish oil, nGSH, or CoQ10 + fish oil supplementation. Levels in the retinal and choroidal homogenates were standardized using total cellular protein levels. The open bars represent the room air (RA) groups and the solid bars represent the IH groups. Data are expressed as mean ± SD (*n* = 6 samples/group).

The levels of Notch-4 in the serum (panel A), retinal homogenates (panel B), and choroidal homogenates (panel C) are presented in Figure 6. Treatments with nGSH and CoQ10 + FO in RA reduced the Notch-4 in the serum, but not with treatment in IH (panel A). In contrast, a significant elevation was seen in the retina with treatment in RA and IH compared with the other treatment groups (panel B). An opposite response was seen in the choroid showing elevations with OO, CoQ10, and FO and reductions with nGSH and CoQ10 + FO in RA and IH, although all treatments resulted in lower levels compared with OO (panel C). The representative Notch-4 expression in the retinal layers showed no significant differences between the treatment groups in RA or IH.

### 2.6. Notch Ligands

**Figure 7:**
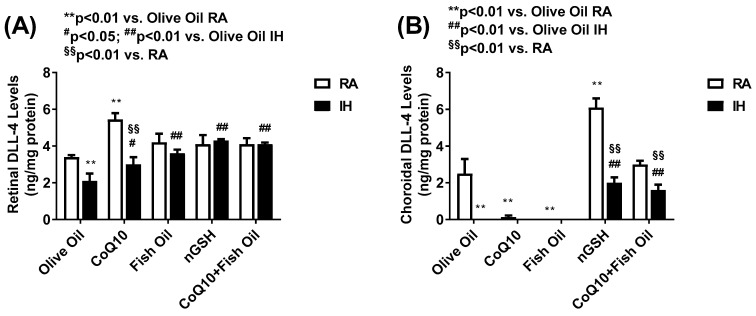
Levels of DLL-4 in the retina (**A**) and choroid (**B**) in response to neonatal IH with olive oil, CoQ10, fish oil, nGSH, or CoQ10 + fish oil supplementation. DLL-4 was not detected in the serum. Levels in the retinal and choroidal homogenates were standardized using total cellular protein levels. The open bars represent the room air (RA) groups and the solid bars represent the IH groups. Data are expressed as mean ± SD (*n* = 6 samples/group).

**Figure 8:**
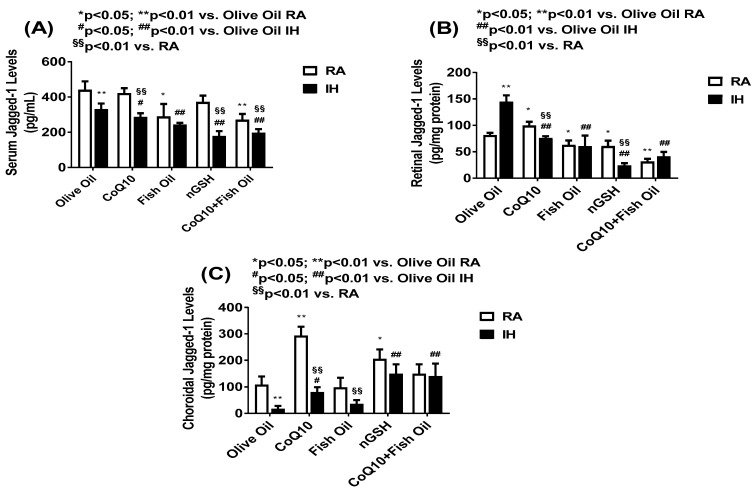
Levels of Jagged-1 in the serum (**A**), retina (**B**), and choroid (**C**) in response to neonatal IH with olive oil, CoQ10, fish oil, nGSH, or CoQ10 + fish oil supplementation. Levels in the retinal and choroidal homogenates were standardized using total cellular protein levels. The open bars represent the room air (RA) groups and the solid bars represent the IH groups. Data are expressed as mean ± SD (*n* = 6 samples/group).

The Notch ligands, namely, DLL-4 and Jagged-1, act via Notch receptors. DLL-4 is the only Notch ligand predominantly expressed by the vascular endothelium. While DLL-4/Notch signaling regulates angiogenesis by remodeling and regression resulting in decreased neovascularization, The blockade of DLL-4/Notch signaling enhances the chaotic, nonproductive vascular sprouting by promoting VEGF-driven vascularization. In contrast, Jagged-1/Notch signaling is proangiogenic and functions by downregulating DLL4–Notch signaling. Figure 7 shows the levels of retinal (panel A) and choroidal (panel B) DLL-4 levels. DLL-4 was not present in the serum. In the retina, CoQ10 produced the highest levels in RA, while all treatments increased the DLL-4 levels in IH compared with OO. In the choroid, only nGSH and CoQ10 + FO increased the DLL-4 levels in RA and IH, although nGSH produced the highest levels in RA. Figure 8 shows the levels of Jagged-1 in the serum (panel A), retina (panel B), and choroid (panel C). In RA and IH, all treatments reduced the Jagged-1 levels compared with OO, and the lowest levels were achieved with nGSH and CoQ10 + FO in IH. Similarly, in the retina, Jagged-1 levels were suppressed with all the treatments in RA and IH compared with OO. Interestingly, exposure to IH produced very high levels of Jagged-1 in the OO group, while treatment with nGSH and CoQ10 + FO was the most effective for its suppression. This coincided with retinal vascular and layer abnormalities. The quantitative analysis results of DLL-4 and Jagged-1 in the retinal layers are presented in Table 2. In RA and IH, DLL-4 was significantly elevated with nGSH and CoQ10 + FO treatment. IH induced Jagged-1 in the OO group and all treatments reduced it, but the most effective was nGSH.

### 2.7. Western Blots

**Figure 9:**
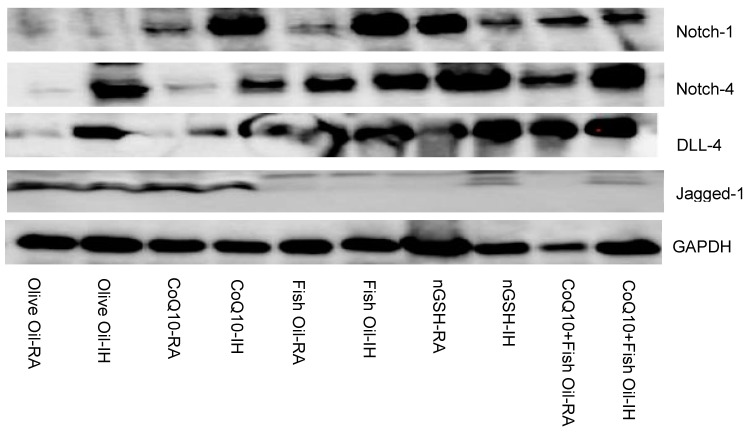
Representative Western blots of GAPDH, Notch-1, Notch-4, DLL-4, and Jagged-1 in the retina of rats at P21. Rats were exposed to neonatal intermittent hypoxia and supplemented with daily oral olive oil, CoQ10, fish oil, nGSH, or CoQ10 + fish oil from postnatal day 0 (P0) to P14, then placed in room air (RA) from P14 to P21 with no further treatment. RA controls were raised in ambient temperature and similarly supplemented.

Figure 9 shows the Western blots of Notch-1, Notch-4, DLL-4, and Jagged-1 in the retina at P21 for the supplemented groups. Similar to the ELISA findings, DLL-4 was induced with FO, nGSH, and CoQ10 + FO, whereas an opposite suppression was seen for Jagged-1. Notch was also induced in all supplemented groups.

### 2.8. VEGF_165_

**Figure 10:**
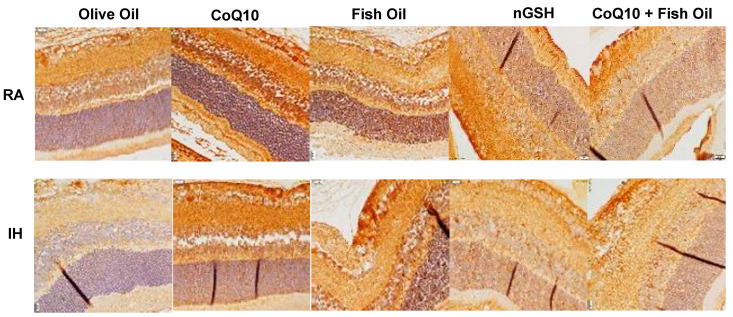
Representative immunoreactivity of VEGF_165_ (brown) in the retinal layers at P21. Images were counterstained with hematoxylin (blue). RA-exposed retinas are represented in the upper panels and IH-exposed retinas are represented in the lower panels. Images show 40× magnification (scale bar is 20 µM).

Representative images of the VEGF_165_ expression (brown stain) in the retinal layers are presented in Figure 10. VEGF_165_ was highly expressed in all layers of the retina, but the highest was seen in the NFL/GCL layer. In RA, treatment with CoQ10 induced VEGF_165_ in all layers. A similar induction was noted with nGSH. The effect of the CoQ10 induction of VEGF_165_ remained sustained in IH. In contrast with RA, treatment with fish oil in IH increased VEGF_165_, while treatment with nGSH reduced it. The corresponding quantitative analysis results are presented in Table 2.

### 2.9. VEGFR-1

**Figure 11:**
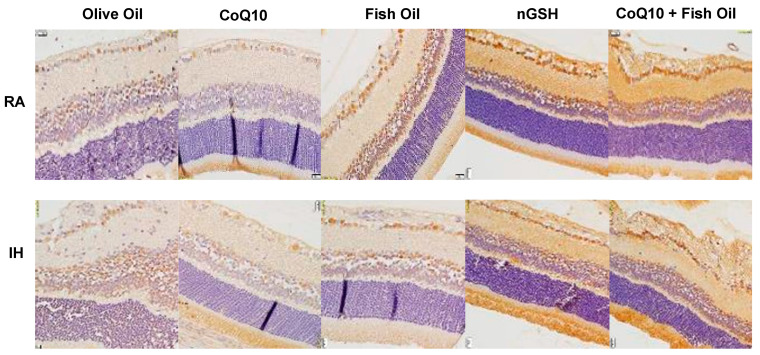
Representative immunoreactivity of VEGFR-1 (brown) in the retinal layers at P21. Images were counterstained with hematoxylin (blue). RA-exposed retinas are represented in the upper panels and IH-exposed retinas are represented in the lower panels. Images show 40× magnification (scale bar is 20 µM).

VEGFR-1 is known to be a VEGF “trap”. It regulates angiogenesis by binding to VEGF, making it less available to VEGFR-2. Representative images of VEGFR-1 expression (brown stain) in the retinal layers are presented in Figure 11. VEGFR-1 was found mostly in the NFL/GCL and INL layers. Treatment with fish oil and, to a greater extent, nGSH and CoQ10 + FO induced VEGFR-1 in both RA and IH. This corresponded to the retinal morphometry presented in Table 1, confirming its inhibitory role. The corresponding quantitative analysis results are presented in Table 2.

### 2.10. Lipid Peroxidation

**Figure 12:**
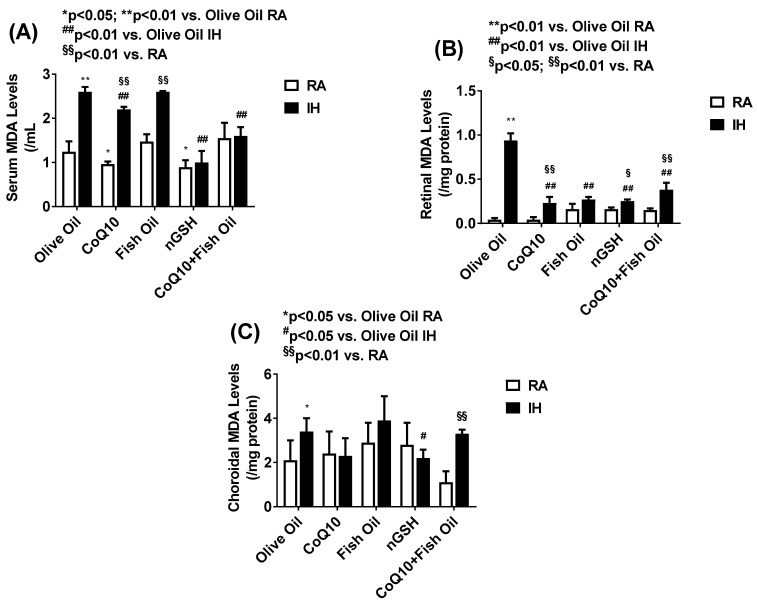
Levels of malondialdehyde (MDA) in the serum (**A**), retina (**B**), and choroid (**C**) in response to neonatal IH with olive oil, CoQ10, fish oil, nGSH, or CoQ10 + fish oil supplementation. Levels in the retinal and choroidal homogenates were standardized using total cellular protein levels. The open bars represent the room air (RA) groups and the solid bars represent the IH groups. Data are expressed as mean ± SD (*n* = 6 samples/group).

Lipids are the major target for reactive oxygen species. Oxidized polyunsaturated fatty acids (PUFAs) can be measured by malondialdehyde (MDA), which is the principal end product of PUFA peroxidation, and is regarded as a valid biomarker of oxidative stress. Levels of MDA in the serum (panel A), retina (panel B), and choroid (panel C) are presented in Figure 12. In the serum, MDA was induced with OO, CoQ10, and FO in IH, and suppressed with nGSH and CoQ10 + FO. In the retina, MDA was also induced in the IH-exposed group treated with OO. All supplements suppressed MDA in IH. In the choroid, only nGSH suppressed MDA in IH.

### 2.11. Nestin

**Figure 13:**
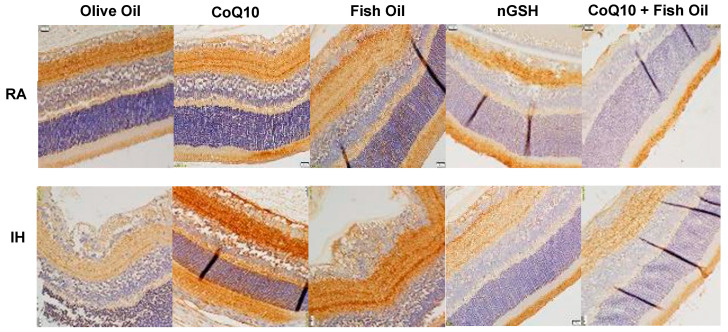
Representative immunoreactivity of nestin (brown) in the retinal layers at P21. Images were counterstained with hematoxylin (blue). RA-exposed retinas are represented in the upper panels and IH-exposed retinas are represented in the lower panels. Images show 40× magnification (scale bar is 20 µM).

Nestin is a biomarker for neural progenitor cells. Representative images of nestin expression (brown stain) in the retinal layers are presented in Figure 13. Nestin was predominantly found in the inner plexiform layer (IPL) and R/C layers. In RA, nestin was induced with FO and suppressed with nGSH and CoQ10 + FO. Nestin was suppressed in IH with OO and induced with CoQ10 and FO. Compared with RA, the CoQ10 + FO combination in IH induced nestin. The corresponding quantitative analysis results are presented in Table 2.

### 2.12. Apoptosis

**Figure 14:**
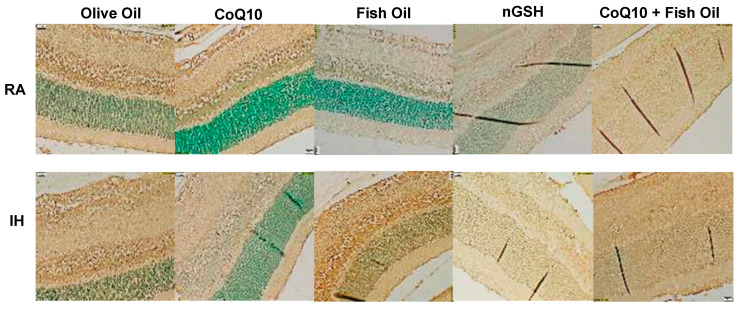
Representative TUNEL (apoptosis)-stained retinas (brown) at P21. Images were counterstained with methyl green (green). RA-exposed retinas are represented in the upper panels and IH-exposed retinas are represented in the lower panels. Images show 40× magnification (scale bar is 20 µM).

Apoptosis in the retinal layers, which is represented by the terminal deoxynucleotidyl transferase dUTP nick-end labeling (TUNEL) stain (brown), is presented in Figure 14. Images were counterstained with methyl green. In RA, FO and nGSH suppressed apoptosis compared with OO. In IH, apoptosis was increased with OO, FO, and CoQ10 + FO. The corresponding quantitative analysis results are presented in Table 2.

### 2.13. HIF_1α_

Representative images of HIF_1α_ expression (brown) in the retinal layers are presented in the Supplementary Materials (Figure S2). HIF_1α_ expression was highest in the NFL/GCL layer in all groups. However, the highest expression was seen in the OO group exposed to IH. All treatments reduced the HIF_1α_ expression in IH compared with OO. The corresponding quantitative analysis results are presented in Table 2.

### 2.14. Eye Opening

The retina of rats develops and matures postnatally. At birth, the rat retina is largely undifferentiated and eye opening concurs with maturation of the retinal neural circuitry, corneal development, and overall maturation of the visual cortex, usually at or around P14. By this time, many retinal cells are mature and the layers are differentiated. For this reason, rats were exposed to neonatal IH until P14. The eyes were assessed daily for signs of opening and the results are presented in the Supplementary Materials (Table S1). In RA, groups treated with FO, nGSH, and CoQ10 + FO predominantly had both eyes opened at P14. Exposure to IH decreased eye opening in all groups, but the highest number of rats with both eyes opened were seen with the group treated with CoQ10 + FO.

### 2.15. Somatic Growth

Due to differences in weight at birth and the presence of runts in some litters, we used the percentage change in body weight and body length from birth to P21. Monitoring the postnatal weight gain is a key non-invasive tool for identifying ROP risk, as poor postnatal growth is an important predictor of ROP. Data are presented in the Supplementary Materials (Table S1). In the RA groups, the highest body weight accretion was seen with nGSH and the lowest with FO, whereas the highest linear growth was seen with FO and nGSH. Overall, exposure to IH reduced weight gain and linear growth in all groups. The highest weight accretion and linear growth was seen with FO and the lowest with OO.

## 3. Discussion

The present study described the changes in the integrity of the retinal vasculature, retinal layers, retinal astrocytic template, and Notch receptors and ligands in response to neonatal IH during retinal development, while at the same time evaluating the effects of supplementation with antioxidants and/or FO on these retinal outcomes in response to neonatal IH. We employed a clinically relevant 50/12% O_2_ IH paradigm to test the hypothesis that supplementation with either antioxidants (CoQ10 or nGSH) and/or FO during neonatal IH can induce DLL-4/Notch while curtailing Jagged-1/Notch, thereby reducing the severity of OIR. This hypothesis was based on previous reports of FO reduction of ROP [34,35,36,37,38,39], severity of OIR [40,41,42], effects on major neonatal diseases [46,47], and the beneficial bioenergetic properties of CoQ10 [48,49,50,51]. The 50/12% O_2_ IH paradigm that was used in our study was previously shown to be associated with severe retinal outcomes [18,52] and closely mimics brief hypoxic episodes experienced by most preterm infants requiring therapeutic oxygen.

Our data show that IH induced abnormalities evidenced by aberrant retinal vasculature and the astrocytic template; furthermore, the histopathology correlated with induction in Jagged-1 and reduction in DLL-4, thus proving our hypothesis. A further confirmation of our hypothesis was the reduction in retinal Jagged-1 with both nGSH and the CoQ10 + FO combination concurrent with induced DLL-4 and Notch. However, given that the combination treatment was not as effective as nGSH for preventing retinal vascular and astrocyte damage or histopathological outcomes, we concluded that nGSH was the superior supplement. While all supplements reduced vascular tortuosity and retinal layer thickness compared with olive oil, nGSH was the most effective for preventing IH-induced vascular architectural abnormalities and significantly reduced the number of endothelial cells violating the vitreous fluid.

In mammals, there are five Notch ligands (DLL-1, DLL-3, DLL-4, Jagged-1, and Jagged-2) and four receptors (Notch-1 to Notch-4) [53]. However, DLL-4 is confined to ECs. The inhibition of DLL-4/Notch signaling promotes excessive sprouting and EC proliferation in the retina [27,45]. The mechanism of DLL-4/Notch signaling inhibition of angiogenesis is via the suppression of VEGFR-2, and possible activation of soluble VEGFR-1. Our data showed that supplementation with both nGSH and the combined CoQ10 + fish oil increased the retinal Notch coincident with DLL-4 levels, suggesting elevated DLL-4/Notch signaling. This finding is important, particularly because the retinas were examined at P21 during re-oxygenation/reperfusion/vasoproliferation. As previously reported [27], DLL-4/Notch signaling inhibits angiogenesis. This was indeed demonstrated by the retinal flatmounts (Figure 1), astrocyte template (Figure 2), and retinal histopathology (Figure 3). Therefore, it seems plausible that early supplementation with nGSH or combined CoQ10 + FO may prevent neovascularization in the developing retina. It was interesting to note the differences between the Notch and DLL-4 levels between the retina and choroid. While there is no definitive explanation for these differences, it may have been due to their inherent structural and function differences. In addition to retinal ECs, the cells in the retina include retinal astrocytes, Müller cells, and tight junctions, while the highly vascularized choroid has large amounts of mast cells and is fenestrated. Nevertheless, both nGSH and the CoQ10 + FO combination induced DLL-4 in the choroid to levels consistent with that of the retina. This may have been due to the effects of the supplements on VEGFR-1.

The relationship between DLL-4, Notch, and VEGFR-1 is less clear. DLL-4 is the most potent Notch ligand regulating angiogenesis. The loss of even one copy of DLL-4 causes extensive angiogenic defects, resulting in embryonic lethality in mice [43,54]. The expression of DLL-4 in tip cells is induced by VEGF, with subsequent activation of Notch on neighboring cells, followed by the downregulation of VEGFR-2 and VEGFR-3. DLL-4/Notch gain-of-function results in a poorly branched vascular network, while DLL-4/Notch loss-of-function causes excessive endothelial cells proliferation, forming a dense, tip-cell-rich, chaotic, and poorly functional vascular network [43,53]. However, while VEGF signaling to VEGFR-2 results in potent angiogenesis, numerous studies have shown that the activation of VEGFR-1 results in an opposite effect. VEGFR-1 acts as a VEGF “trap”, thus making VEGF unavailable to VEGFR-2, and thus, plays a negative role in angiogenesis [55]. Indeed, our data show that both nGSH and CoQ10 + FO induced VEGFR-1, similar to Notch and DLL-4. This finding is corroborated by previous reports that Notch increases VEGFR-1 [56] and provides key insights into the mechanism of how these supplements may regulate angiogenesis. Our findings provide the first evidence for antioxidants and/or FO regulation of DLL-4/Notch/VEGFR-1 in retinal angiogenesis, and establish a functional link between antioxidants, FO, and the DLL-4/Notch/VEGFR-1 signaling pathways.

In contrast, Jagged-1 signaling to Notch has been shown to have the opposing effects by promoting endothelial cell proliferation and sprouting angiogenesis and inhibits DLL-4/Notch signaling in endothelial cells [25]. Jagged-1 deletion results in a decrease in retinal vascular density and causes suppression of the tip cell phenotype, whereas Jagged-1 overexpression opposes DLL-4 to promote sprouting [57]. Our findings of elevated retinal Jagged-1 in the IH-exposed retinas of rats supplemented with OO were correlated with the retinal abnormalities and OIR in that group, thus confirming previous reports. It was also noteworthy that all supplements suppressed Jagged-1 in RA and IH, but the most effective supplement was nGSH. Taken together, nGSH and the combined CoQ10 + FO treatment demonstrated a significant reduction in IH-induced OIR correlating with the induction of DLL-4/Notch and simultaneous inhibition of Jagged-1/Notch, providing evidence for a potentially new, non-invasive therapeutic intervention for OIR.

Considering the high levels of lipids in the retina and the susceptibility of PUFAs to lipid peroxidation, we examined levels of MDA in the systemic and ocular compartments, as well as apoptosis in the retinal layers. We noted that IH significantly induced MDA levels in the serum, retina, and choroid, but the levels were reduced with nGSH and CoQ10 + FO in the serum and with all supplements in the retina. These findings are in harmony with previous reports [58,59], and further demonstrate the harmful effects of neonatal IH on lipids. Our data not only support the use of antioxidants in combination with lipids but also the use of nGSH to prevent the adverse effects of neonatal IH. While this study has clinical relevance, there were limitations. First, we studied the effects of neonatal IH during the reoxygenation/reperfusion phase at P21 and did not examine the retina immediately post IH exposure, prior to the placement of the rats in RA. Second, since OO itself may have antioxidant properties, we did not have a group with the nGSH and FO combination that could have compared the potency of two antioxidants for preventing FO lipid peroxidation. Third, we did not examine all Notch and receptor isoforms and focused mainly on those isoforms related to retinal angiogenesis. Finally, the IH model used in these experiments, although similar, did not exactly replicate the arterial oxygen desaturations experienced by ELGANs, who require oxygen therapy for chronic lung disease. Despite these limitations, our data provide important clues regarding some of the underlying mechanisms associated with angiogenesis of the developing retina exposed to neonatal IH and possible benefits of antioxidants with or without FO.

## 4. Materials and Methods

### 4.1. Animals

All experiments were approved by the State University of New York (SUNY), Downstate Health Sciences University Institutional Animal Care and Use Committee, Brooklyn, NY. The animals were treated humanely according to the guidelines outlined by the United States Department of Agriculture and the Guide for the Care and Use of Laboratory Animals. Certified infection-free, timed-pregnant Sprague Dawley rats were purchased from Charles River Laboratories (Wilmington, MA, USA) at 18 days gestation. The animals were housed in an animal facility with a 12 h day/12 h night cycle and provided standard laboratory diet and water ad libitum until the delivery of their pups. All procedures were performed in accordance with the Association for Research in Vision and Ophthalmology statement on the Use of Animals in Ophthalmic and Vision Research.

### 4.2. Experimental Design

Within 2–4 h of birth, newborn rat pups delivered on the same day were pooled to eliminate litter differences (4 litters) and randomly assigned to expanded litters of 18 pups/litter (9 males and 9 females). Gender was identified by the anogenital distance. The expanded litter size was used to simulate poor nutrition and relative postnatal malnutrition of ELGANs who are at increased risk for severe ROP. Animals were exposed to neonatal IH from P0 to P14, then allowed to recover in RA until P21. During IH, pups were administered daily oral doses of (1) CoQ10 (0.35 mg in 50 µL extra virgin olive oil (OO)) purchased from Sigma Aldrich (St. Louis, MO, USA); (2) 50 µL fish oil (FO) containing 35 mg total *n*-3 PUFAs (22 mg eicosapentaenoic acid (EPA) and 13 mg docosahexaenoic acid, (DHA)), where the doses of *n*-3 PUFAs and CoQ10 were based on results of our previous findings [40,41]; (3) glutathione nanoparticles (nGSH) sublingual drops (200 mg/mL, optimized for instant absorption) diluted to 24 µg in 50 µL with extra virgin OO, where the dose of nGSH was based on the manufacturer’s recommended dose for a 70 kg adult (Nanoceutical Solutions, San Antonio, TX, USA) and on previous findings showing that nGSH was safe, and resulted in improved absorption, delivery, and blood concentrations of GSH [60]; (4) combined oral CoQ10 + FO (50 µL); or (5) 50 µL extra virgin OO (control). RA littermates were raised in atmospheric oxygen, were similarly supplemented, and served as age-matched controls. At P14, the pups were placed in RA until P21 with no further supplementation. A representation of the experimental design is presented in the Supplementary Materials (Figure S1). The body weight and linear growth (crown to rump length) and the time of eye opening (cecal period in rats representing maturation of the retinal neural circuitry) were recorded.

### 4.3. Neonatal Intermittent Hypoxia (IH) Profile

Animals randomized to neonatal IH were placed with the dams in specialized oxygen chambers attached to an oxycycler (BioSpherix, Parish, NY, USA). The IH profile consisted of an initial exposure of hyperoxia (50% O_2_) for 30 min, followed by three brief 1 min, clustered hypoxic events (12% O_2_), with a 10 min reoxygenation in 50% O_2_ between each hypoxic event. Recovery from IH occurred in 50% O_2_ following each clustered IH event for 2.5 h for a total of 8 clustering IH episodes per day for 14 days, as previously described [17,18,40,41,42]. Oxygen saturation was confirmed on a sentinel unanesthetized rat pup from each group using the MouseOx Pulse Oximeter and WinDaq Waveform Browser software ver. 2.0 (STARR Life Sciences Corp., Oakmont, PA, USA) before and after the IH exposure.

### 4.4. Sample Collection and Processing

At P21, mixed arterial and venous blood samples were collected following decapitation into sterile Eppendorf tubes and placed on ice for 30 min. Samples were centrifuged at 3500 rpm for 30 min at 4 °C. The resulting serum was transferred to a clean sterile Eppendorf tube and frozen at −20 °C until analysis. All samples were analyzed on the same day. A total of 6 serum samples per group (3 males and 3 females) were analyzed for MDA, Notch-1, Notch-4, DLL-4, and Jagged-1. For the retinal histopathology and immunohistochemistry (IHC), whole heads with eyes in situ were placed into 10% neutral-buffered formalin (NBF). Once fixed, whole eyes were enucleated and placed in embedding cassettes for processing, staining, and mounting on slides for histopathology (Histowiz, Inc., Long Island City, NY, USA). Unstained slides were used for the IHC. For the ADPase-stained retinal flatmounts, the eyes were enucleated and rinsed in ice-cold phosphate buffered saline (PBS, pH 7.4) on ice and placed in 4% paraformaldehyde (PFA, pH 7.4) on ice. For the enzyme-linked immunosorbent assay (ELISA) and Western blot (WB) assays, the eyes were enucleated and rinsed in ice-cold PBS on ice. The retinal and choroidal tissues were obtained by careful dissection from the sclera under a dissecting microscope. Samples were pooled for a total of 4 samples per group (2 males and 2 females). The samples were placed in sterile Lysing Matrix D 2.0 mL tubes containing 1.4 mm ceramic spheres (MP Biomedicals, Santa Ana, CA, USA) and snap-frozen in liquid nitrogen. Samples were stored at −80 °C until analysis. All the samples were analyzed on the same day. On the day of analyses, the tubes were allowed to defrost on ice and placed in a high-speed FastPrep-24 instrument (MP Biomedicals, Santa Ana, CA, USA) for homogenization in sterile PBS (ELISA) or RIPA lysis buffer (WB).

### 4.5. Retinal Flatmounts

Eyes were enucleated and placed in 4% PFA on ice. The eyes were placed in the refrigerator for 90 min, after which they were placed in ice-cold PBS on ice. Following the removal of the cornea and lens, the retina was separated from the sclera, cut in 4 quadrants, and flattened. Retinas dedicated for ADPase staining were immersed in 4% PFA and stored overnight at 4 °C. Retinas dedicated for glial fibrillary acidic protein (GFAP) and isolectin B4 double staining were immersed in PBS/Triton X-100 (TXPBS) purchased from Boston BioProducts, Ashland, MA, USA.

### 4.6. ADPase Staining of the Retinas

After 24 h of incubation in 4% PFA, the retinas were washed in tris maleate buffer (pH 7.2) on ice prior to incubation in ADPase incubation medium containing 3.0 mM lead nitrate and 6.0 mM magnesium chloride (Sigma Chemical Co., St. Louis, MO, USA) in tris maleate buffer (pH 7.2). After incubation, the retinas were washed with tris maleate buffer prior to the addition of diluted ammonium sulfide (Fisher Scientific, Silver Spring, MD, USA) for 1 min. The retinas were washed again in tris maleate buffer and flat-mounted on a microscope slide with PBS and glycerin. All images were captured using an Olympus BX53 microscope at 10× magnification (scale bar is 100 μm), DP72 digital camera, and CellSens Dimension imaging software (version 2.1) from Olympus America, Inc. (Center Valley, PA, USA), attached to an HP Z44 computer.

### 4.7. GFAP and Isolectin B4 Staining

GFAP and isolectin B4 double staining of the retinal flatmounts were conducted as previously described [17,18,40,41]. Briefly, retinal flatmounts were washed in ice-cold PBS/Triton X-100 (TXPBS) and fixed in methanol, followed by permeabilization and blocking in PermBlock (PBS + 0.3% Triton X-100 + 0.2% bovine serum albumin) in 5% goat serum for 1 h. After washing in TXPBS, flatmounts were incubated with rabbit GFAP primary antibody (Cell Signaling Technologies, Danvers, MA, USA) overnight at 4 °C. Following several washes with TXPBS, the flatmounts were incubated with Alexa Fluor 488 goat anti-rabbit fluorescent secondary antibodies and Alexa Fluor 594 Isolectin B4 (ThermoFisher Sci/Life Technologies, Grand Island, NY, USA) overnight at 4 °C. The flatmounts were washed with TXPBS and mounted on slides with *prolong* anti-fade fluorescent mounting media and imaged at 20× magnification (scale bar is 50 μm) using the Olympus BX53 microscope, DP72 digital camera, and CellSens Dimension imaging software (version 2.1) from Olympus America, Inc. (Center Valley, PA, USA), attached to an HP Z44 computer.

### 4.8. Retinal Angiogenesis and Morphometric Analyses

The tortuosity index, vessel diameter, and number of endothelial cells present in the nerve fiber layer (NFL)/ganglion cell layer (GCL) were used to determine retinal angiogenesis, as previously described [17,61]. All measurements were conducted using the CellSens software ver. 1.18 (Olympus America, Inc., Center Valley, PA, USA). The diameter of the arteries and the veins was measured around the optic nerve from the optic disk to the first branch using the arbitrary line tool. The tortuosity of the vessels was quantified by tracing a line along the tortuous vessel using the polyline tool and comparing the length of the tortuous vessel to the length of the straight vessel from the optic disk to the first branch using the arbitrary line tool. The number of endothelial cells present in a defined area of 1000 µm^2^ was determined using the count and measure tool. Measurements for the total retinal thickness and thickness of each retinal layer corresponding to the nerve fiber layer/ganglion cell layer (NFL/GCL), inner plexiform layer (IPL), inner nuclear layer (INL), outer plexiform layer (OPL), outer nuclear layer (ONL), and rods and cones (R/Cs) were used for the morphometric analyses using the arbitrary line tool of the CellSens software, ver. 1.18.

### 4.9. Assay of MDA

All supplements were lipid based, therefore we determined levels of lipid peroxidation. Lipid peroxidation is the reaction of oxygen with unsaturated lipids that produces oxidation products. One of the main primary products of lipid peroxidation is MDA, which is the most mutagenic product of lipid peroxidation, and has been extensively used as a biomarker for lipid peroxidation [62]. The levels of MDA in the serum and retinal and choroidal homogenates were determined using commercially available kits purchased from Millipore Sigma (St. Louis, MO, USA). All samples were processed and assayed according to the manufacturer’s protocol. Retinal and choroidal levels were standardized using total cellular protein levels.

### 4.10. Assay of Notch Ligands and Receptors

Notch signaling is activated upon cell-to-cell contact as a result of interactions between Notch receptors and their ligands (DLL or Jagged). Notch signaling regulates many biological functions, such as apoptosis, cell proliferation, differentiation, and lineage decisions during embryonic development [63,64]. In the retina, DLL-4 signaling to Notch acts as a negative regulator on VEGF-mediated angiogenesis, while Jagged-1 signaling to Notch promotes potent angiogenesis that antagonizes DLL-4 mediated Notch signaling [27,65]. The levels of Notch-1, Notch-4, DLL-4, and Jagged-1 in the serum and the retinal and choroidal homogenates were determined using commercially available activity assay kits purchased from MyBioSource (San Diego, CA, USA). Samples were processed and assayed according to the manufacturer’s protocol. Retinal and choroidal levels were standardized using the total cellular protein levels.

### 4.11. Total Cellular Protein Levels

On the day of assays, an aliquot (10 µL) of the retinal and choroid homogenates was utilized for total cellular protein levels using the Bradford method (Bio-Rad, Hercules, CA, USA) with bovine serum albumin as a standard.

### 4.12. IHC

Formalin-fixed tissue sections (5 microns) were deparaffinized in a series of xylenes and graded alcohols, followed by washing in de-ionized water. After unmasking the antigens using sodium citrate buffer, pH 6.0, sections were washed in Tris-buffered saline with Tween 20 (TBST) and incubated in a humidified chamber for 1 h at room temperature in blocking buffer (Cell Signaling Technology, Danvers, MA, USA). After the removal of the blocking serum, sections were incubated overnight with primary antibodies for HIF_1α_ (MyBioSource, San Diego, CA, USA), VEGF_165_ (Antibodies Online, Limerick, PA, USA), Notch-1, Notch-4, DLL-4, and Jagged-1 (Novus Biologicals, Centennial, CO, USA) at 4 °C. The sections were washed with TBST and incubated with secondary antibody SignalStain Boost Detection Reagent (Cell Signaling Technology). After washing with TBST, sections were incubated with SignalStain DAB chromogen (Cell Signaling Technology) and counterstained with hematoxylin, followed by dehydration in ethanol and xylene, then mounted with SignalStain mounting medium (Cell Signaling, Danvers, MA, USA). Images were captured at 40× magnification using the Olympus BX53 microscope, DP72 digital camera, and CellSens Dimension imaging software (version 2.1) from Olympus America, Inc. (Center Valley, PA, USA), attached to an HP Z44 computer.

### 4.13. Western Blots

All samples were analyzed on the same day. On the day of the assay, 400 µL ice-cold RIPA lysis buffer (BioRad, Hercules, CA, USA) was added to the tubes containing the retina or choroid tissue samples. The samples were homogenized in a high-speed FastPrep-24 instrument (MP Biomedicals, Santa Ana, CA, USA). After the addition of an additional 200 µL ice-cold RIPA lysis buffer to each tube, the samples were agitated for 2 h at 4 °C in an orbital shaker, then centrifuged at 12,000 rpm for 20 min at 4 °C. The protein content of the supernatant was determined using the BioRad protein assay and all samples were adjusted to 5 mg/mL protein. After the addition of equal volumes of 2× Laemmli buffer containing 2-mercaptoethanol, the samples were boiled for 5 min at 95 °C, then loaded (15 µL) onto mini Protean TGX precast gels (BioRad, Hercules, CA, USA) for electrophoresis in tris-glycine/SDS buffer, pH 8.3. The proteins were then transferred to Trans Blot transfer membranes using the Trans Blot Turbo machine (BioRad) and confirmed using Ponceau S solution. After blocking the membrane with 1% TBS with casein (BioRad), Notch-1, Notch-4, DLL-4, and Jagged-1 primary antibodies (1:10,000 dilution) were added. The membranes were incubated with gentle agitation at 4 °C overnight and washed in TBST prior to the addition of horseradish peroxidase (HRP)-conjugated secondary antibodies (1:10,000 dilution) and incubation for 1 h. The membranes were washed and the detection substrate consisting of luminol and peroxide (1:1 ratio) added. The membranes were imaged using the ChemiDoc Imaging system (BioRad).

### 4.14. Statistical Analysis

To determine the differences between the supplemented groups within each oxygen environment, a test for normality was first conducted using the Bartlett’s test. Normally distributed data were analyzed using two-way analysis of variance (ANOVA) with Dunnett’s post hoc tests. Non-normally distributed data were analyzed using the Kruskal–Wallis test with Dunn’s multiple comparison test. To determine the differences between the RA and IH groups for each supplement, a test for normality was first conducted using the Levene’s test for equality of variances. Normally distributed data were analyzed using the unpaired *t*-test and non-normally distributed data were analyzed using the Mann–Whitney U test. Data are presented as mean ± SEM and a *p*-value of <0.05 was considered as statistically significant using SPSS version 16.0 (SPSS Inc., Chicago, IL, USA). Graphs were prepared using GraphPad Prism version 7.03 (GraphPad, San Diego, CA, USA).

## 5. Conclusions

In conclusion, given that the currently available treatment for severe ROP targeting the very vulnerable preterm newborn infant is fraught with multiple barriers and challenges, the overarching goal of this descriptive study was to identify a more age-appropriate, non-invasive, effective alternative therapy for ROP. The model used in these experiments was repeatedly shown to produce significant OIR with characteristics similar to severe ROP. We showed for the first time that there is a beneficial synergy between antioxidants and FO for inducing the DLL-4/Notch/VEGFR-1 pathway to curtail aberrant angiogenesis. We also showed that exposure to neonatal IH induced Notch/Jagged-1 to promote neovascularization and retinal damage, and supplementation with nGSH or CoQ10 + FO reversed this effect. The question remains regarding why the two supplements produced such similarities. At this point, we can only speculate that CoQ10 prevents FO lipid peroxidation, and together, they can produce similar effects to glutathione, which is the master antioxidant. Nevertheless, considering the retinal vascular and retinal histopathological outcomes, the most effective supplement was nGSH. Clinical trials are needed to establish whether infants at high risk for severe ROP are haploinsufficient for DLL-4 and whether supplementation with nGSH is a potential therapy.

## Figures and Tables

**Table 1:** Retinal morphometry.

	Olive Oil	CoQ10	Fish Oil	nGSH	CoQ10 + Fish Oil
Room Air (RA)					
Tortuosity index	0.96 ± 0.05	0.97 ± 0.05	0.98 ± 0.03	1.0 ± 0.02	1.0 ± 0.03
Artery diameter (µm)	55.3 ± 9.3	50.6 ± 11.8	46.1 ± 7.3	27.6 ± 0.69 *	26.6 ± 0.63 *
Vein diameter (µm)	41.9 ± 10.3	38.4 ± 9.8	32.2 ± 5.9	40.6 ± 1.5	43.8 ± 1.2
No. ECs in NFL/GCL layer	181.1 ± 16.6	173.0 ± 16.6	176.1 ± 12.8	211.0 ± 28	166.8 ± 14.9
Total retinal thickness (µm)	269.3 ± 6.8	248.2 ± 5.0	343.3 ± 11.6 **	261.6 ± 9.2	303.0 ± 9.0 *
NFL/GCL thickness (µm)	34.1 ± 8.1	29.5 ± 1.8	50.9 ± 4.1 *	26.8 ± 3.1	41.8 ± 3.0
IPL thickness (µm)	53.3 ± 1.6	47.9 ± 0.84	67.2 ± 4.0 **	51.6 ± 2.3	48.1 ± 3.7
INL thickness (µm)	57.7 ± 0.98	48.3 ± 1.2	59.8 ± 2.3	43.4 ± 3.4 **	52.9 ± 4.2
OPL thickness (µm)	14.7 ± 0.7	14.1 ± 0.56	17.6 ± 0.7 **	12.4 ± 0.74	14.5 ± 0.63
ONL thickness (µm)	81.6 ± 1.1	71.4 ± 1.4 *	82.8 ± 2.4	73.7 ± 2.9	87.3 ± 4.8
R/C thickness (µm)	37.1 ± 1.1	32.5 ± 1.3	45.5 ± 2.3 **	32.0 ± 2.6	40.1 ± 1.8
Intermittent Hypoxia (IH)					
Tortuosity index	1.52 ± 0.24 ^§§^	1.1 ± 0.1 ^##^	1.08 ± 0.04 ^##^	1.03 ± 0.03 ^##^	1.08 ± 0.08 ^##^
Artery diameter (µm)	60.9 ± 3.4	45.1 ± 12.2	42.7 ± 10.3	29.5 ± 0.57 ^##,§^	29.2 ± 0.65 ^##,§§^
Vein diameter (µm)	48.3 ± 8.3	37.6 ± 5.9	34.9 ± 7.3	41.6 ± 0.95	44.2 ± 1.03
No. ECs in NFL/GCL layer	639.5 ± 148.4 ^§§^	274.2 ± 32.7 ^#,§§^	610.3 ± 81.5 ^§§^	300.0 ± 41.9 ^#^	770.3 ± 73.1 ^§§^
Total retinal thickness (µm)	379.4 ± 10.6 ^§§^	330.8 ± 11.7 ^##,§§^	308.6 ± 13.1 ^##,§^	307.0 ± 16.7 ^##,§^	286.7 ± 11.5 ^##^
NFL/GCL thickness (µm)	72.4 ± 7.9 ^§§^	49.2 ± 4.0 ^##,§§^	41.2 ± 2.7 ^##,§^	42.0 ± 3.4 ^##,§§^	54.1 ± 4.6^#,§^
IPL thickness (µm)	63.0 ± 2.2 ^§§^	61.0 ± 2.4 ^§§^	52.3 ± 2.5 ^§§^	61.0 ± 5.1	46.0 ± 2.8 ^##^
INL thickness (µm)	57.5 ± 1.5 ^§§^	73.4 ± 2.4 ^§§^	59.1 ± 4.2	59.5 ± 3.5 ^§§^	49.9 ± 2.2 ^##^
OPL thickness (µm)	18.1 ± 0.65 ^§§^	17.7 ± 3.6 ^§§^	15.6 ± 0.6 ^§§^	16.6 ± 1.6 ^§§^	16.5 ± 0.82
ONL thickness (µm)	94.1 ± 2.3 ^§§^	90.8 ± 5.1 ^§§^	109.5 ± 7.0 ^§§^	86.6 ± 2.9 ^§§^	95.4 ± 7.2
R/C thickness (µm)	58.2 ± 2.7 ^§§^	44.9 ± 2.0 ^##,§§^	48.0 ± 4.0 ^#^	40.7 ± 1.9 ^##,§§^	40.2 ± 1.9 ^##^

CoQ10 (coenzyme Q10), nGSH (glutathione nanoparticles), RA (room air), ECs (endothelial cells), NFL (nuclear fiber layer), GCL (ganglion cell layer), IPL (inner plexiform layer), INL (inner nuclear layer), OPL (outer plexiform layer), ONL (outer nuclear layer), and R/C (rod and cone). Data are mean ± SEM (*n* = 12–36 measurements per group). * *p* < 0.05 and ** *p* < 0.01 vs. olive oil in RA; ^#^ *p* < 0.05 and ^##^ *p* < 0.01 vs. olive oil in IH using one-way ANOVA with Dunnett’s post hoc multiple comparison test; ^§^ *p* < 0.05 and ^§§^ *p* < 0.01 RA vs. IH using unpaired *t*-test.

**Table 2:** Retinal quantitative immunohistochemistry intensities.

	Olive Oil	CoQ10	Fish Oil	nGSH	CoQ10 + Fish Oil
*Room Air (RA)*					
Notch-1	12,221.9 ± 404.6	14,501.7 ± 378.6 *	14,784.6 ± 379.2 **	23,151.2 ± 808.7 **	18,245.5 ± 750.7 **
DLL-4	6915.3 ± 306.9	3325.1 ± 159.9 **	2317.1 ± 86.9 **	16,473.9 ± 696.9 **	16,907.0 ± 681.2 **
Jagged-1	12,481.7 ± 744.8	12,722.4 ± 347.3	19,277.8 ± 600.1 **	12,166.4 ± 684.0	12,517.7 ± 619.6
GFAP	26,370.4 ± 555.9	20,405.6 ± 433.6 **	21,796.3 ± 604.1 **	20,876.6 ± 1027.7 **	28,311.1 ± 1290.8
HIF_1α_	3114.1 ± 351.1	3028.8 ± 190.6	2061.0 ± 304.8 *	681.8 ± 49.6 **	2794.4 ± 446.7
VEGF_165_	9395.5 ± 284.4	20,770.9 ± 1146.3 **	15,096.4 ± 799.1 **	14,043.8 ± 900.5 **	5879.1 ± 984.0 **
VEGFR-1	2549.6 ± 112.0	1939.7 ± 77.6	3644.7 ± 199.4	9991.0 ± 631.3 **	7485.3 ± 357.4 **
Nestin	17,562.0 ± 514.2	20,229.1 ± 578.9	13,373.8 ± 854.5 **	13,690.9 ± 900.9 **	9490.6 ± 1007.6 **
Apoptosis	2629.1 ± 319.5	2855.0 ± 188.8	745.8 ± 124.0 **	524.9 ± 72.4 **	1321.6 ± 151.6 **
*Intermittent Hypoxia (IH)*					
Notch-1	14,804.0 ± 379.5 ^§§^	13,479.1 ± 554.3	14,482.5 ± 748.9	15,798.2 ± 367.7 ^§§^	17,638.2 ± 912.7 ^##,§§^
DLL-4	5749.8 ± 336.1^§^	3028.5 ± 133.5 ^##^	2829.8 ± 214.1 ^##,§^	13,985.6 ± 527.2 ^##,§§^	1480.9 ± 678.4 ^##,§§^
Jagged-1	19,913.3 ± 217.1 ^§§^	14,091.6 ± 401.8 ^##§^	15,960.2 ± 821.8 ^##,§§^	12,341.0 ± 542.4 ^##,§§^	15,610.7 ± 362.4 ^##,§§^
GFAP	22,055.9 ± 336.0 ^§§^	25,644.4 ± 604.3 ^#,§§^	22,582.2 ± 766.8	19,288.8 ± 1562.6	18,498.6 ± 951.3 ^#^
HIF_1α_	7694.6 ± 444.6 ^§§^	1353.7 ± 321.0 ^##,§§^	4740.5 ± 449.3 ^##,§§^	1851.3 ± 113.7 ^##,§§^	4395.5 ± 566.7 ^##,§^
VEGF_165_	14,909.4 ± 793.0 ^§§^	18,180.6 ± 817.8 ^##^	18,207.6 ± 548.5 ^##,§§^	5175.2 ± 521.3 ^##,§§^	5031.7 ± 331.6 ^##^
VEGFR-1	1932.7 ± 81.5 ^§^	2051.9 ± 219.1 ^§§^	1186.9 ± 57.5 ^§§^	9246.0 ± 490.9 ^##^	7213.0 ± 445.6 ^##^
Nestin	15,755.4 ± 560.5 ^§§^	25,224.1 ± 682.9	14,154.7 ± 1081.7	14,856.1 ± 464.3	10,295.0 ± 656.2 ^##^
Apoptosis	3696.1 ± 204.9 ^§§^	2920.5 ± 181.7	2882.2 ± 241.3 ^§§^	1914.9 ± 216.8 ^##,§§^	3785.8 ± 332.2 ^§§^

DLL-4 (delta-like ligand)-4, HIF (hypoxia inducible factor), VEGF (vascular endothelial growth factor), VEGFR (vascular endothelial growth factor receptor), GFAP (glial fibrillary acidic protein), CoQ10 (coenzyme Q10), and nGSH (glutathione nanoparticles). Data are mean ± SEM (*n* = 12–36 measurements per group). * *p* < 0.05 and ** *p* < 0.01 vs. olive oil in RA; ^#^ *p* < 0.05 and ^##^ *p* < 0.01 vs. olive oil in IH using one-way ANOVA with Dunnett’s post hoc multiple comparison test; ^§^ *p* < 0.05 and ^§§^ *p* < 0.01 RA vs. IH using unpaired *t*-test.

## Data Availability

The data that support the findings of this study are available from the corresponding author upon reasonable request.

## Supplementary Materials

The following supporting information can be downloaded from https://www.mdpi.com/article/10.3390/ph17030381/s1. Figure S1: Experimental design; Figure S2: Representative immunoreactivity of GFAP (brown) in the retinal layers at P21; Figure S3: Representative immunoreactivity of HIF_1α_ (brown) in the retinal layers at P21; Table S1: Eye opening and somatic growth.
