# Supplementary material for: Comparison of Glutathione Nanoparticles, CoEnzyme Q10, and Fish Oil for Prevention of Oxygen-Induced Retinopathy in Neonatal Rats
Source: Pharmaceuticals (Basel). 2024 Mar 17;17(3):381. doi: 10.3390/ph17030381 (PMC10975314; doi:10.3390/ph17030381)
Supplement: Supplementary file 1 [file pharmaceuticals-17-00381-s001.zip › pharmaceuticals-2861470-supplementary.pdf]

Table S1: Eye Opening and Somatic Growth

Group	Eye Opening at P14 (both eyes)	% Change in Body Weight from P0	% Change in Body Length from P0
Room Air (RA):			
Olive Oil	7 (39%)	513.0±13.5	88.8±1.5
CoQ10	7 (39%)	479.7±18.8	87.6±1.8
Fish Oil	16 (89%)**	422.7±13.8**	99.0±2.5*
nGSH	16 (89%)**	563.6±17.1	114.2±3.8**
CoQ10+Fish Oil	16 (89%)**	456.2±21.2	93.8±2.7
Intermittent Hypoxia (IH):			
Olive Oil	0 (0%)§§	319.9±12.3§§	66.2±2.2§§
CoQ10	6 (33%)#	304.5±10.3§§	86.7±2.5##
Fish Oil	10 (56%)##§	499.4±19.3##§§	106.9±1.9##§
nGSH	6 (33%)##§§	390.8±18.6##§§	92.6±1.8##§§
CoQ10+Fish Oil	15 (83%)##	398.9±13.1##§	84.9±1.7##§§

CoQ10 (Coenzyme Q10), nGSH (glutathione nanoparticles), P0 (birth, postnatal day 0). Data are mean±SEM (n=18 rats per group). *p<0.05, **p<0.01 vs Olive Oil in RA; #p<0.05, ##p<0.01 vs Olive Oil in IH. Eye opening was analyzed using 2-sided Fisher's exact test. % change in body weight and length were analyzed using one-way ANOVA with Dunnett's post hoc multiple comparison test for comparison among the treatment groups in each oxygen environment and comparison between RA and IH was analyzed using unpaired t-test (§p<0.05, §§p<0.01).

Figure S1 – Experimental Design

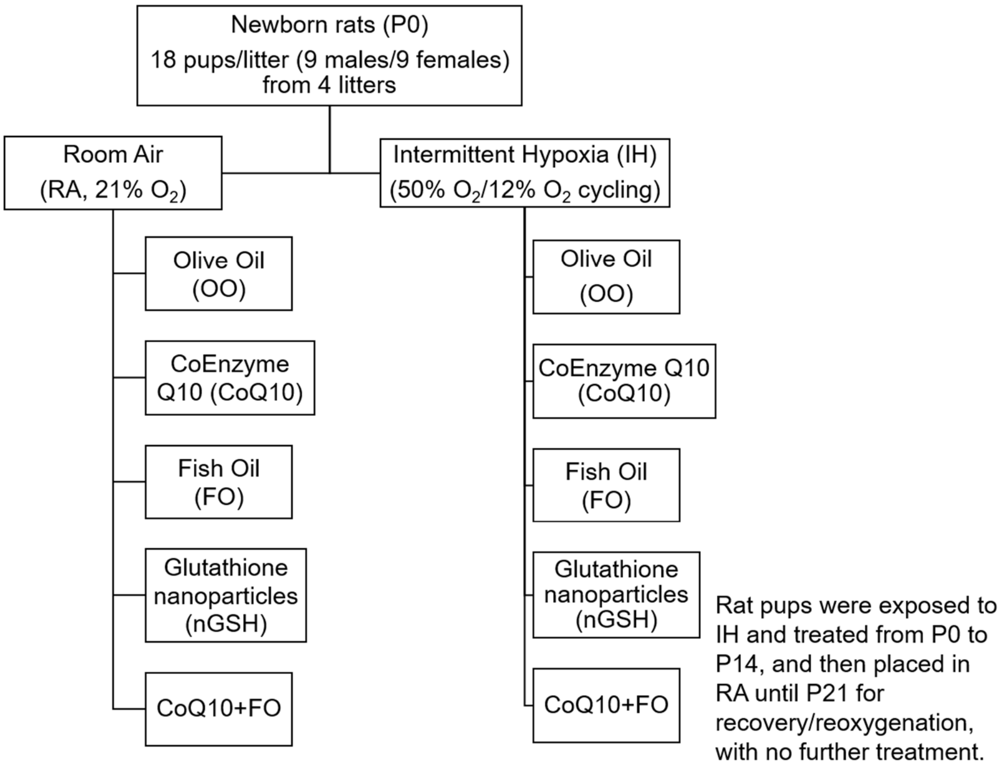

Rat pups were exposed to IH and treated from P0 to P14, and then placed in RA until P21 for recovery/reoxygenation, with no further treatment.

Figure S2 – GFAP

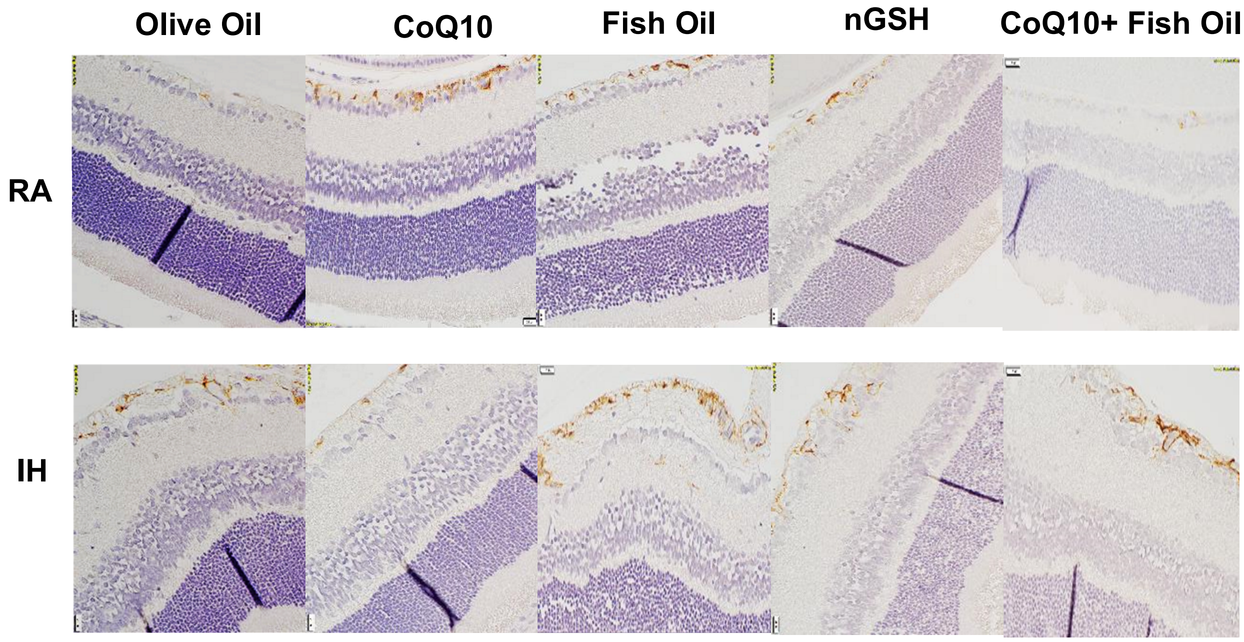

Figure S2. Representative immunoreactivity of GFAP (brown) in the retinal layers at P21. RA-exposed retinas are represented in the upper panels and IH-exposed retinas are represented in the lower panels. Images are 40X magnification (scale bar is 20 µM). GFAP was present only in the NFL/GCL layer.

Figure S3 - HIF$_{1α}$

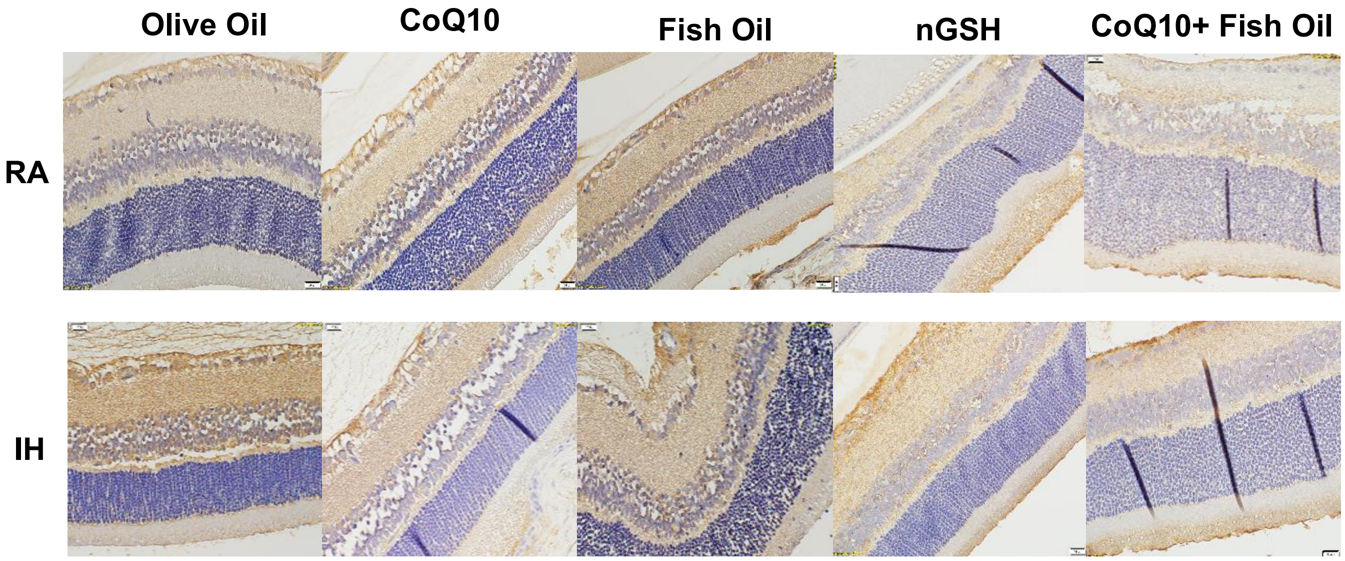

Figure S3. Representative immunoreactivity of HIF$_{1α}$ (brown) in the retinal layers at P21. RA-exposed retinas are represented in the upper panels and IH-exposed retinas are represented in the lower panels. Images are 40X magnification (scale bar is 20 μM).
